# Oxidative stress in pediatric diseases associated with the origin of life and growth and development

**DOI:** 10.3389/fcell.2025.1550765

**Published:** 2025-07-15

**Authors:** Bo Zheng, Jianhua Fu

**Affiliations:** ^1^ Department of Pediatrics, Shenyang Women’s and Children’s Hospital, Shenyang, China; ^2^ Department of Pediatrics, Sheng Jing Hospital of China Medical University, Shenyang, China

**Keywords:** oxidative stress, pediatric diseases, fetal diseases, antioxidants, free radicals

## Abstract

The presence of oxidative stress and an imbalance in antioxidant mechanisms have been demonstrated in numerous diseases. Furthermore, mounting evidence suggests that the occurrence, progression, and prognosis of certain pediatric diseases linked to the origin of life and growth and development are also associated with oxidative stress. In this review, we systematically analyze the relationship between oxidative stress and various pediatric diseases, proposing new theoretical foundations and therapeutic targets for their treatment.

## 1 Introduction

**FIGURE 1:**
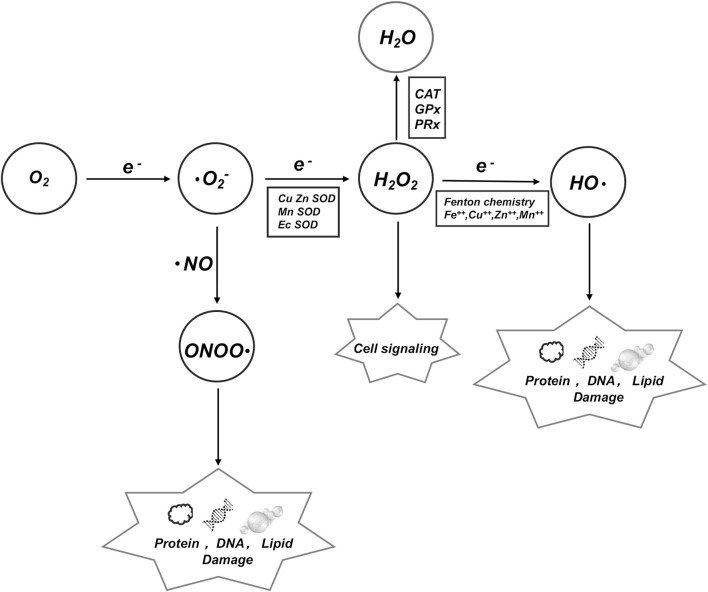
ROS is an umbrella term for an array of derivatives of molecular oxygen which includes superoxide anion (^.^ O_2_), hydroxyl radical (.OH) and hydrogen peroxide (H_2_O_2_), etc. We demonstrate the conversion mechanisms among various key factors and their impacts on DNA, proteins, and lipids.

Oxidative stress (OS) happens when there is an uneven balance between harmful oxidative processes and protective antioxidants in the body. This leads to inflammation, more neutrophils, extra proteases being released, and a lot of oxidative substances being produced. Oxidative stress is a deleterious effect caused by free radicals within the body and is regarded as a crucial factor in aging and disease. The most significant contributors to oxidative stress are reactive oxygen species (ROS), have established that oxidative stress plays a role in numerous adult diseases, such as Cancer, chronic obstructive pulmonary disease (COPD) and Alzheimer’s disease influencing their onset, progression, and even overall prognosis hydroxyl radical (.OH), hydrogen peroxide (H_2_O_2_), and (.O_2_^−^) etc. (Sies and Jones, 2020) (Figure 1). Previous researches have established that oxidative stress plays many important roles in numerous adult disease systems, such as the nervous system, respiratory system, digestive system, etc. (Valko et al., 2007). Recent evidence suggests that OS is also related to children’s growth and pediatric diseases (Table 1). Thus, we will focus on the latest advancements in understanding the molecular properties of OS in select pediatric diseases linked to the origin of life and the processes of growth and development.

**TABLE 1:** List and supporting evidence of oxidative stress-related fetal and pediatric diseases.

Diease	Test specimen	Antioxidant stress indicators	Ref
IUGR	Maternal urine	8-oxodG +	Potdar et al. (2009)
	Infant serum	d-ROM +	Ashina et al. (2021)
		BAP -	
	Plasma newborns	Plasma total antioxidant activity (ORAC)-,VC-,VE-	Saker et al. (2008)
		Hydroperoxide and carbonyl protein levels +	
	Umbilical cord arterial blood	MDA+; SOD-;catalase +; reduced glutathione +	Dede et al. (2017), Gupta et al. (2004)
GDM	Rat embryonic cells	GSH-	Trocino et al. (1995)
	Maternal placenta	8-isoprostane+	Coughlan et al. (2004), Lappas et al. (2004)
	cord arterial blood and placenta	MDA+; GSH+; SOD-	Kinalski et al. (2001)
	Maternal placenta	Nrf2+; catalase +; SOD1+	Manoharan et al. (2019)
	Maternal placenta	apolipoprotein D (apo D) +	Navarro et al. (2010)
CHDs	Mouse myocardial cells	eNOS+	Feng et al. (2002)
	Mice	SOD1 Reversed 149 miRNAs that can cause CHDs	Dong et al. (2016)
	Mice	NAC ∼ GSH+∼ROS- ∼ CHDs-	Moazzen et al. (2014)
	Mice	SOD1∼wnt+∼CHD-	Wang et al. (2015a), Wang et al. (2015b)
TTTS	Ewes	In pregnant ewes bearing twin foetuses:GSH-;GSH-Px -;MDA +	Gur et al. (2011)
	Pregnant women’s peripheral blood	In twin pregnancies:TBARS+; CAT-;VC-	Jantsch et al. (2020)
	Fetal cord blood and amniotic fluid	In smaller twins Mt DNA-	Chang et al. (2013)
BPD	Mice	c-Abl +	Singleton et al. (2009)
	Mice	NADPH oxidase (NOX 1 +)	Carnesecchi et al. (2009)
	Infant peripheral blood	MnSOD -	Asikainen and White (2004), Berkelhamer and Farrow (2014)
	Infant peripheral blood	GSSG/GSH +	Vento et al. (2009)
NEC	Swiss webster mice pups; rat intestinal epithelial	H2O2 +; IGF-1--H2O2 -	Baregamian et al. (2011)
	Human fetal intestinal epithelial cells		
	Cord blood of preterm newborns	TH +; AOPP+; NPBI +	Perrone et al. (2010)
	Plasma newborns	Total oxidant status (TOS) +; Oxidative stress index (OSI) +	Aydemir et al. (2011)
	Mice	NOD-like receptor+; TLR-4 +	Li et al. (2021), Zhao et al. (2018)
HIE	Rat	lipoxin A4 (LXA4) -; LXA4--IκB/NF-κB pathway	Zhu et al. (2020)
	Zebrafish	RNA (lncRNA) LINC00938 -; SH-SY5Y -	Zhao et al. (2023)
		NACA-- LINC00938+	
	Rat	Myricetin-- Nrf2+--ROS-	Chen et al. (2023)
	Mice	Echinocystic acid (EA)--PI3K/Akt/Nrf2 +--ROS-	Li et al. (2023)
IVH	Rabbit	cyclooxygenase-2(COX-2)+	Vinukonda et al. (2010)
	Rabbit	Nitrotyrosine +; 4-hyroxynonenal +; 8-OHdG +	Zia et al. (2009)
		O2·− +; H2O2 +	
		apocynin--NAD(P)H - --ROS -	
	Human multicenter clinical survey	Mitochondrial quantity +	Chang et al. (2017)
ROP	Mice	NO synthase +	Wang et al. (2013)
	Mice	NAD(P)H oxidase +	Al-Shabrawey et al. (2005)
	Mice	eNOS +	Brooks et al. (2001), Beauchamp et al. (2004)
	Bovine retinal endothelial cells	Nitric oxide synthase (NOS3) +; peroxynitrite +	Gu et al. (2003)
Obesity	Children’s serum	Total oxidant status (TOS) +, total anti-oxidant status (TAS) +	Kilic et al. (2016)
	Children’s plasma, erythrocytes, and urine	TBARS +	Lechuga-Sancho et al. (2018)
	Children’s plasma	Isoprostane +	Correia-Costa et al. (2016)
	Children’s plasma	retinol-; β-carotene -;vitamin E −	Stenzel et al. (2018)
Diabetes	TIDM-Children’s serum	Cu/Zn +	Rychert-Stos et al. (2022)
	TIDM-Children’s serum	Cu/Zn +; MDA+; total antioxidant status (TAS) -	Grabia et al. (2023)
	T2DM-Children’s serum	G6PD -	Mahmoud and Nor El-Din (2013), Heymann et al. (2012)
	T2DM-Chang liver cells	mitochondrial OS +	Handy et al. (2009)
GHD	Human fibroblasts	SIRT1 -	Haigis and Sinclair (2010), Yamamoto and Takahashi (2018)
	Mice	SIRT1 -	Shtaif et al. (2020)

In this table, we show the changes of some indicators related to oxidative stress in the nutrition and endocrine related diseases of fetuses, newborns and children, as well as the species origin of the tested samples; (+) representing the increased expression level of corresponding indicators in diseases; (−) represents the reduction of the expression level of the corresponding indicators in the disease.

## 2 Oxidative stress in fetal diseases

**FIGURE 2:**
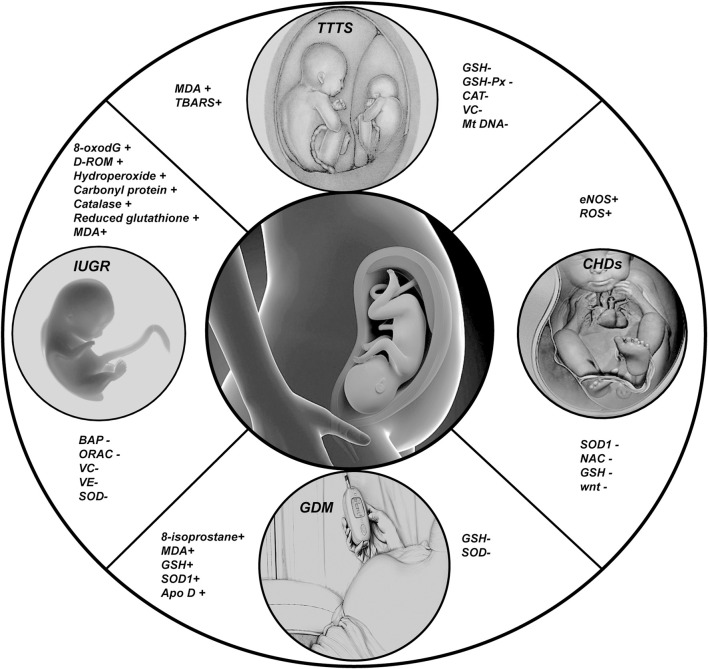
In this Figure we show the changes of some important indicators of fetal oxidative stress related diseases; (+) representing the increased expression level of corresponding indicators in diseases; (−) represents the reduction of the expression level of the corresponding indicators in the disease.

The fetal period is a critically important phase in the early stages of life, and diseases occurring during this period can significantly impact individual health during early postnatal and even mature stages. Recent studies have shown that various fetal diseases are closely associated with oxidative stress events (Figure 2).

### 2.1 Intrauterine growth restriction (IUGR)

IUGR refers to a condition where fetal size does not reach its genetic growth potential *in utero*. Specifically, fetal birth weight is classified as being at a weight that is two standard deviations lower than the average weight for the same age of development or falling below the 10% of the typical weight for that age. Adverse exposures during pregnancy, such as air pollution, smoking, and malnutrition, can increase oxidative activity in pregnant women, leading to a significant rise in endogenous ROS levels in the placenta (Geca et al., 2022). Reports have linked the concentration of 8-oxo-7,8-dihydro-20-deoxyguanosine (8-oxodG) in the urine of mothers at 12 weeks of gestation with an increased risk of IUGR (Potdar et al., 2009), indicating that oxidative stress may occur before the symptoms of IUGR are noticeable. Consistent with previous reports, Ashina et al. (2021) fended that in children with IUGR, the levels of reactive oxidative derivatives, specifically d-ROM, are elevated, while the biological antioxidant potential (BAP) is diminished. In cases of IUGR related to maternal malnutrition, concentrations of antioxidants in the plasma of both mothers and newborns are significantly lower, contrasted by elevated levels of oxidants (Saker et al., 2008; Dede et al., 2017; Gupta et al., 2004). Moreover, mitochondrial swelling was observed in endothelial cells derived from IUGR cells cultured *in vitro* (Formanowicz et al., 2019).

### 2.2 Gestational diabetes mellitus (GDM)

GDM is a short-term condition where the body has trouble with carbohydrate metabolism, high blood sugar levels, insulin resistance, and insufficient insulin secretion or effectiveness,during pregnancy (ACOG Practice Bulletin, 2018). GDM is closely linked to neonatal hypoglycemia and the developmental disorders of fetal pulmonary surfactant. Studies have shown that pregnant women with GDM experience a noticeable rise in lipid peroxidation and oxidative stress levels in placental tissues compared to normal control groups (Trocino et al., 1995; Coughlan et al., 2004; Lappas et al., 2004). Interestingly, unlike other conditions, during GDM, as oxidative stress products rise, there is also an increase in antioxidant enzymes within the placenta (Coughlan et al., 2004; Madazli et al., 2008; Kinalski et al., 2001; Chaudhari et al., 2003). This may suggest that there is a possible defense system against antioxidants in the placenta that is connected to nuclear factor erythroid 2-related factor 2 (Nrf2). Activation of the Nrf2/antioxidant response element (ARE) pathway leads to heightened expression of SOD1 and other antioxidant enzymes (Manoharan et al., 2019). Additionally, apolipoprotein D (apo D) may also play an important role in the antioxidant defense system of the placenta in GDM. Reports indicate that the levels of apo D are significantly increased in trophoblastic and villous cells surrounding large blood vessels in GDM placental tissue compared to controls (Navarro et al., 2010).

Neural tube defects (NTDs) are a group of common and devastating congenital malformations that appear in early pregnancy due to the disturbance of normal neural tube closure. Studies on the underlying mechanism of diabetic maternal embryopathy have shown that oxidative stress is a major factor in the formation of NTDs (Chang TI. et al., 2003; Yang et al., 2008; Matough et al., 2012). The excessive apoptosis of cells caused by oxidative stress may be one of the important mechanisms that induce deformities (Marino et al., 2014).

### 2.3 Congenital heart defects (CHDs)

CHDs are the most prevalent structural anomalies at birth, occurring in 1%–5% of live births (Pierpont et al., 2007; Gilboa et al., 2016; Benjamin et al., 2018). This category includes conditions such as ventricular septal defects and patent ductus arteriosus; while they may not show obvious signs in the early stages, the underlying issues can progressively worsen. CHDs account for a significant portion of pediatric mortality in developed countries (Cleves et al., 2003). The heart, being the first fully functional organ developed during embryonic growth, is guided by the interaction of conserved transcription factors responsible for growth, morphogenesis, and contractility (DeRuiter et al., 1992). The onset of CHD is more closely associated with nitric oxide (NO) signaling pathways. Nitric oxide synthase (NOS) facilitates the conversion of L-arginine to nitric oxide through NADPH-dependent reactions within the cellular context. Endothelial nitric oxide synthase (eNOS), one of the three NOS isoforms that typically binds to cell membranes, is responsive to changes in intracellular calcium concentration and is predominantly expressed in endothelial and myocardial cells (Knowles and Moncada, 1994). Studies reveal that eNOS is expressed at high levels in embryonic heart cells, underscoring its critical role in cardiogenesis (Liu and Feng, 2012; Feng et al., 2002). In murine models, eNOS expression was detected in embryonic cardiac structures at 9.5 weeks of gestation, peaking at 13.5 weeks and subsequently declining (Liu and Feng, 2012). Vitro studies have indicated that NOS inhibitors can elicit the differentiation of embryonic stem cells into cardiomyocytes (Bloch et al., 1999). Furthermore, research has documented that ROS regulates key genes and miRNAs that influence heart development during fetal growth (Moazzen et al., 2015; Dong et al., 2016). Further investigation of the fetal heart indicates that disrupting ROS levels by overexpressing agents such as N-acetylcysteine or the SOD1 gene can effectively reduce ROS levels, consequently decreasing the incidence of CHDs (Moazzen et al., 2014; Wang et al., 2015a; Wang et al., 2015b).

Thoracic aortic aneurysms (TAA) are abnormal aortic dilatations and a major cardiovascular complication of Marfan syndrome (MFS). Previous studies have shown that oxidative stress is the main cause of TAA (Budbazar et al., 2023; Phillippi et al., 2010; Asano et al., 2022).

### 2.4 Twin-to-twin transfusion syndrome (TTTS)

TTTS is a rare complication that occurs in 10%–15% of monochorionic multiple pregnancies. TTTS primarily arises from an imbalance in oxygen and nutrient supply due to placental vascular anastomoses between twins (Denbow et al., 2000; Quintero, 2003). Research findings indicate that mothers carrying twins exhibit a significantly increased expression of lipid peroxidation markers malondialdehyde (MDA) etc. In peripheral blood and placental tissues compared to those with singleton pregnancies (Gur et al., 2011; Jantsch et al., 2020). These studies highlight the pivotal role of lipid peroxidation in TTTS. Furthermore, evidence suggests that oxidative stress can elevate mitochondrial DNA (mtDNA) levels, thereby impacting fetal growth (Lee et al., 2000). Analogous to findings in twin studies, elevated mtDNA content has been observed in smaller gestational age fetuses, further substantiating the role of oxidative stress in TTTS (Chang et al., 2013).

## 3 Oxidative stress in preterm infants

**FIGURE 3:**
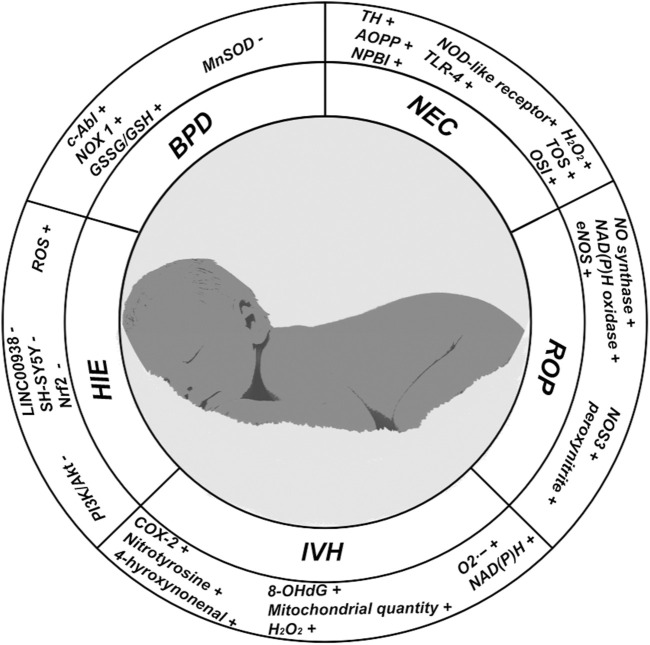
In this figure, we show the changes of some important indicators in neonatal oxidative stress-related diseases; (+) representing the increased expression level of corresponding indicators in diseases; (−) represents the reduction of the expression level of the corresponding indicators in the disease.

Preterm infants face significant challenges. In recent years, advancements in neonatal intensive care unit (NICU) techniques have led to a gradual decline in mortality and long-term disability rates among preterm infants. However, the influence of increasing oxidative stress on the emergence and progression of various systemic diseases in preterm infants is gathering increasing attention (Figure 3).

### 3.1 Bronchopulmonary dysplasia (BPD)

BPD is a chronic lung disease that affects premature infants, particularly those born extremely preterm (before 28 weeks of gestation). Infants with BPD experience severe impairment of oxygen transport and diffusion capabilities within the alveoli (Nordlund et al., 2017). As early as 1967, the Northway team first identified high oxygen exposure as a major risk factor contributing to BPD (Northway, 1967). Recent studies have indicated that exposure to any concentration of oxygen within the first few hours of life can provoke oxidative stress, potentially heightening the risk of BPD (Vento et al., 2009). The determination of an appropriate oxygen concentration for mechanically ventilated preterm infants in early life remains contentious (Saugstad, 2001). Datta et al. (2015); Nardiello et al., 2017) demonstrated through mouse models that oxygen exposure at any concentration during the initial stages can stunt or even impair alveolar development. High oxygen concentrations can directly damage alveoli, particularly alveolar type II epithelial cells, which are critical to both alveolar development and repair (Nabhan et al., 2018; Budinger et al., 2011). Even after the removal of high oxygen exposure, the functional recovery of alveolar type II epithelial cells remains challenging (Ilizarov et al., 2001). Singleton et al. (2009) validated through mouse models that high oxygen environments can damage alveolar epithelial cells and elevate their ROS production. Further research shows that this increase in ROS production is facilitated by elevated NADPH oxidase levels in hypoxic conditions, leading to damage of alveolar epithelial cells (Carnesecchi et al., 2009; Wang et al., 2007). The limited and imbalanced antioxidant defense mechanisms are also pivotal factors contributing to oxidative stress damage in preterm BPD. Mitochondrial or manganese SOD (MnSOD) functions to convert superoxide radicals (O_2_·^-^) into molecular oxygen (O_2_) and hydrogen peroxide (H_2_O_2_). O_2_·^-^ can facilitate the formation of hydroxyl radicals (·OH) through the Fenton reaction, whereas H2O2 decomposes into water and O2 via catalase (CAT) and glutathione peroxidase, effectively mitigating oxidative damage (Ighodaro and Akinloye, 2019). The expression capacity of MnSOD is notably impaired in premature infants, rendering them more susceptible to ROS during high oxygen exposure (Asikainen and White, 2004; Berkelhamer and Farrow, 2014). The balance between reduced glutathione (GSH) and oxidized glutathione (GSSG) serves as a critical component of intracellular antioxidant systems involved in regulating redox states and scavenging free radicals. In preterm infants, initial exposure to hyperoxia elevates the GSSG/GSH ratio, which persists through the early days of life and correlates with worse prognoses for BPD (Vento et al., 2009).

### 3.2 Necrotizing enterocolitis (NEC)

NEC arises from various factors damaging the intestinal mucosa, with an incidence rate of approximately one in ten extremely preterm infants (Hackam and Caplan, 2018; Neu and Walker, 2011; Thompson and Bizzarro, 2008). Characteristic X-ray findings show cystic air accumulation within the intestinal wall. Baregamian et al. (2011) reported that ROS may be the main cause of apoptosis in intestinal epithelial cells through a rat NEC model. The sensitivity of small intestinal epithelial cells in newborns, especially premature infants, to high oxygen is extremely high. Exposure to high concentrations of oxygen can lead to weakened barrier function in the small intestine, destruction of tight junction structures (Wang et al., 2023), and a decrease in Paneth cells (Underwood, 2012; McElroy et al., 2013), as well as increased invasion of harmful bacteria that disrupt the normal function of the small intestinal wall (Wang et al., 2023). Investigations by Perrone et al. (2010); Aydemir et al. (2011) identified potential biomarkers for oxidative stress risk in umbilical cord blood, including biomarkers associated with oxidative stress injuries, such as total hydroperoxides (TH) and advanced oxidation protein products (AOPP) and non-protein-bound iron (NPBI; basal superoxide anion, BSA; stimulated superoxide anion, USSA),with NEC correlating significantly with heightened cord blood levels of NPBI, AOPP, and TH. Liu et al. (2021) found that increased ROS can affect intestinal cell outcomes and functions by altering the covalent states of NO, leading to further dysregulation. Under conditions of hyperoxia, intestinal ROS significantly increases, promoting inflammatory cascades and facilitating the onset of inflammatory bowel disease (Li et al., 2021; Zhao et al., 2018).

### 3.3 Hypoxic-ischemic encephalopathy (HIE)

HIE also referred to as hypoxic-ischemic brain damage (HIBD), is a common cause of mortality among infants, especially those born prematurely (Dixon et al., 2012). Emerging research indicates that the pathogenesis of HIE involves mechanisms such as iron deficiency, inflammation, autophagy, cell necrosis, and apoptosis, with oxidative stress representing a critical component (Zhu et al., 2020; Chen et al., 2021). Zhao et al. (2023) validated using a zebrafish model for HIE that increased ROS production Inhibiting the expression of long-chain-non-coding RNA (lncRNA) LINC00938, leading to mitochondrial dysfunction in SH-SY5Y cells and propelling the progression of HIE. In support of this, pre-treatment with the ROS inhibitor N-acetylcysteine amide (NACA) effectively countered oxidative stress and mitochondrial dysfunction induced by LINC00938 knockout, consequently reducing cellular apoptosis. Furthermore, studies by Chen et al. on rats indicated that myricetin, a naturally extracted flavanol compound, can mitigate apoptosis and oxidative stress via the signaling pathway of Nrf2, offering protective effects against HIE damage (Chen et al., 2023). Li et al. (2023). Validated in neonatal mouse models that Echinocystic acid (EA), a natural plant extract, improves apoptosis and oxidative stress accompanied by activation of the PI3K/Akt/Nrf2 signaling pathway, alleviating hypoxic-ischemic brain damage (HIBD).

### 3.4 Intraventricular hemorrhage (IVH)

IVH is one of the most common neurological diseases in premature infants, affecting tens of thousands of infants worldwide each year (Courtney et al., 2002; Horbar et al., 2002). Survivors of IVH often experience neurodevelopmental disorders, which can include impairments in motor function, cognition, speech, hearing, and vision. It is estimated that approximately 15% of IVH survivors develop cerebral palsy, while 27% exhibit moderate to severe neurosensory disorders by the age of 18–24 months (Quintero, 2003). The occurrence of IVH promotes the activation of microglia, which, in conjunction with activated macrophages, stimulates the release of various ROS, reactive nitrogen species, chemokines and pro-inflammatory cytokines, thereby inducing inflammation and oxidative damage (Vinukonda et al., 2010; Zia et al., 2009). Mesenchymal stem cells (MSCs), which are the most commonly utilized cells in clinical experimental research, also play a role in reducing ROS during the pathological process of IVH (Luyt et al., 2020; van Velthoven et al., 2014). Studies have reported that MSCs can enhance vascular regeneration in areas affected by intracranial hemorrhage and increase the number of mitochondria in undamaged regions (Chang et al., 2017).

### 3.5 Retinopathy of prematurity (ROP)

ROP is frequently observed in premature infants and those with low birth weight, and it represents a common cause of long-term visual impairment and even blindness in this population (Tran et al., 2018; Alajbegovic-Hali et al., 2015; Kim et al., 2018a). High concentration oxygen supply during non-invasive or invasive assisted ventilation with atmospheric oxygen inhalation is a globally recognized exposure factor for ROP. Due to the imperfect retinal blood regulatory system in premature infants, a high oxygen state can produce excessive levels of superoxide to promote the progression of pathological processes (Rivera et al., 2017; Buonocore et al., 2002; Hellstrom et al., 2001). Interestingly, recent studies have indicated that hypoxia can activate nitric oxide synthase and nicotinamide adenine dinucleotide phosphate oxidase, enzymes responsible for generating ROS, which are implicated in oxygen-induced retinopathy (Wang et al., 2013; Al-Shabrawey et al., 2005; Brooks et al., 2001). Furthermore, research indicate that nitro-oxidative stress damage plays a key role in microvascular degenerative diseases such as ROP (Rivera et al., 2017). Under high oxygen conditions, NO production increases and its activity are enhanced. NO and ROS undergo a series of reactions, resulting an increase in the levels of nitrate, nitrite, and peroxide, which subsequently damage microvasculature (Beauchamp et al., 2004; Gu et al., 2003). The antioxidant effects of vitamin E have been widely investigated in the prevention and treatment of ROP. Although the precise therapeutic mechanisms of vitamin E in addressing oxidative stress associated with ROP remain unclear, polymorphisms in the cytochrome P450 4F2 gene have been shown to positively influence vitamin E metabolism (Tsang et al., 2019).

## 4 Oxidative stress in childhood nutrition and endocrine system diseases

**FIGURE 4:**
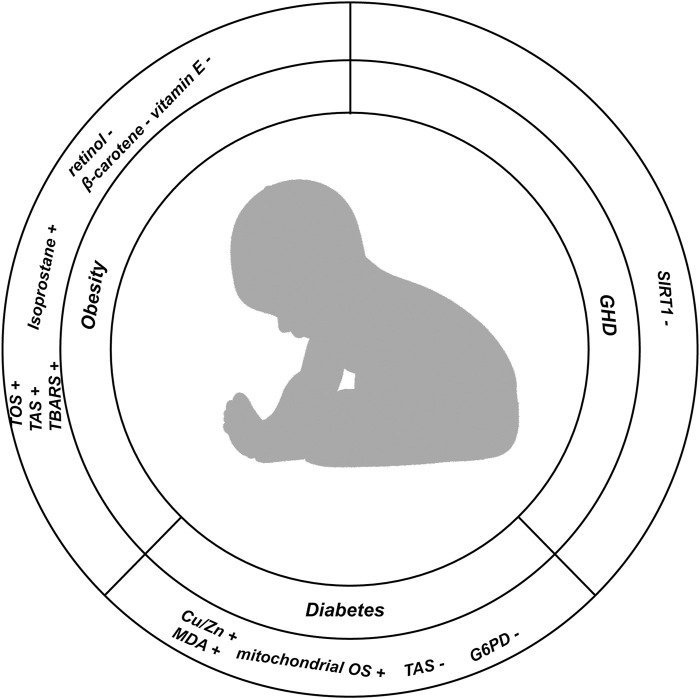
In this Figure, we show the changes of indicators related to oxidative stress in childhood nutrition and endocrine diseases; (+) representing the increased expression level of corresponding indicators in diseases; (−) represents the reduction of the expression level of the corresponding indicators in the disease.

The status of children’s nutrition and endocrine system diseases is crucial for their growth, development, and long-term quality of life. Recent researches have indicated that OS is a significant factor in both the onset and progression of nutritional and endocrine diseases in children (Figure 4).

### 4.1 Obesity

Obesity is defined as the excessive accumulation of fat, which may be an early risk factor for cardiovascular, cerebrovascular diseases, diabetes, and other health issues in adulthood. With the increasing prevalence of obesity among children year by year, childhood obesity has emerged as one of the significant concerns which influence public health. Increasing evidence suggests that OS plays a critical role in the pathological processes associated with childhood obesity. Kilic et al. (2016) found that both total oxidants and antioxidant capacity were elevated in obese children. Lechuga Sancho et al. (Lechuga-Sancho et al., 2018) reported that a marker of lipid peroxidation, thiobarbituric acid reactive substances (TBARS), were significantly elevated in obese children compared to those in the normal control group. This finding also indirectly supports the notion of compromised catalase activity in obese children. Similar results were reflected in the research of Correia Costa et al., which indicated that another lipid peroxidation marker, isoprostane, was markedly upregulated in obese children and correlated with HOMA-IR (Homeostatic Model Assessment for Insulin Resistance), high-sensitivity CRP, urinary H_2_O_2_ and triglyceride levels (Correia-Costa et al., 2016). Furthermore, antioxidants such as β-carotene, vitamin E and retinol were found to be significantly reduced in obese children (Stenzel et al., 2018). Another noteworthy study demonstrated that the percentage of body fat and waist circumference in adolescents is inversely related to their total antioxidant capacity (Leo et al., 2016).

### 4.2 Diabetes

Diabetes is a metabolic disorder characterized by impaired insulin biological effects or defective insulin secretion, or a combination of both. The characteristics of T1DM are insufficient insulin secretion and abnormal blood glucose levels, which are caused by autoimmune damage to pancreatic beta cells (ElSayed et al., 2023). Insulin and zinc ions coexist in the vesicles of pancreatic β-cells, which play a pivotal role in the regulation of insulin secretion (Zysk et al., 2018). Copper ions, as essential cofactors for numerous enzymes, including superoxide dismutase (SOD), are critical for enzyme activity (Jomova et al., 2022). The study by Rychert-Stos et al. (2022); Grabia et al., 2023). Demonstrated that in the body a positive correlation between oxidative stress levels and Cu/Zn ratios, indicating that higher Cu/Zn values are associated morbidity of T1DM in children. Despite T1DM being more prevalent in children, the incidence of T2DM among the pediatric population is rising, coinciding with improvements in global living standards and dietary patterns. The overall incidence of T2DM among children aged 10–14 years in the United States is 8.1 per 100,000, while the total incidence among adolescents aged 15–19 years is 11.8 per 100,000, with the highest rates observed among Native American populations (Mayer-Davis et al., 2018; Pulgaron and Delamater, 2014). There is credible evidence suggesting that the incidence of T2DM in children is expected to increase by approximately 50% in the coming decades, potentially leading to significant increases in cardiovascular and cerebrovascular diseases among adults and imposing substantial burdens on social economies (Imperatore et al., 2012). Glucose-6-phosphate dehydrogenase (G6PD) is one of the key antioxidant enzymes, which reduces nicotinamide adenine dinucleotide phosphate (NADP+) to NADPH. This reaction is a crucial step in the pentose phosphate pathway (PPP) and it plays a key role in the pathological processes associated with T2DM (Ge et al., 2020). Deficiency of G6PD is associated with T2DM, with further studies revealing increased G6PD activity accompanied by decreased levels of the oxidative stress marker HbA1c in T2DM patients (Mahmoud and Nor El-Din, 2013; Heymann et al., 2012). Existing studies have established a strong relationship between insulin resistance and mitochondrial oxidative stress in childhood (Handy et al., 2009). Additionally, ample evidence suggests that mitochondrial oxidative stress can exacerbate the pathological process of T2DM (Victor et al., 2011). In treating adult T2DM, insulin therapy has become a widely recognized approach; however, in pediatric T2DM patients, insulin therapy has not shown significant effects on insulin resistance (Consortium, 2018). Redox omics may offer a key strategy for the treatment of pediatric T2DM in the future (Alu et al., 2022).

### 4.3 Growth hormone deficiency (GHD)

A child’s height falls below the average height of Lower than the average height of healthy children of the same race, gender, and age more than two standard deviations or below the 3rd percentile of the growth curve for normal children is referred to as short stature. Among various factors leading to short stature, growth hormone (GH) secretion by the anterior pituitary gland significantly influences body height. The short stature resulting from a deficiency in GH is termed growth hormone deficiency (GHD), which is also referred to as pituitary dwarfism. GHD is one of the prevalent endocrine disorders in pediatric clinical practice, mostly occurring sporadically, though a small proportion may be inherited. The growth and development of children are regulated not only by GH but also by insulin-like growth factor 1 (IGF-1). IGF-1 is secreted by the liver, which plays a pivotal role in mediating GH function. IGF-1 serves as the primary peripheral mediator of GH. Traditionally, GH regulation is understood to be a balance between growth hormone-inhibiting hormone (GHIH, somatostatin) and growth hormone-releasing hormone (GHRH, somatoliberin), which together form a diurnal secretion pattern in the hypothalamus (Ranke and Wit, 2018; Bonnefont et al., 2005). In recent years, novel regulatory factors have been identified, which may elucidate the mechanisms governing GH secretion and its effects; one such factor is sirtuin 1 (SIRT1) (Haigis and Sinclair, 2010; Yamamoto and Takahashi, 2018). Research has demonstrated that SIRT1 is involved in the osteogenic processes of cartilage and skeletal growth through its regulatory mechanisms (Shtaif et al., 2020). Moreover, SIRT1 participates in multiple important cellular processes, including cell cycle regulation, DNA repair, and apoptosis, with oxidative stress response being a critical area of regulation (Chen et al., 2020; Lee et al., 2019). SIRT1 can modulate the development of adipose tissue, skeletal muscle, and liver by influencing FOXO1, a key factor in oxidative stress that regulates insulin sensitivity (Cao et al., 2016).

## 5 Antioxidant stress therapy

The management of oxidative stress during the perinatal period primarily targets neonatal diseases, focusing on treatment strategies involving enzymes such as MnSOD, CuZnSOD, GSH, ecSOD, and vitamins such as E and A. Trace elements like selenium or L-arginine are used as essential cofactors for these enzymes. However, the application of MnSOD, CuZnSOD, and ecSOD in high-oxygen models in rabbit, rat, and human lung epithelial cells has demonstrated promising results in reducing oxidative stress markers and enhancing alveolar epithelial cell function; nonetheless, the clinical applicability of these therapies is restricted by biochemical and physiological factors [ (Chang et al., 2003b; Padmanabhan et al., 1985; Davis et al., 1985; Koo et al., 2005). In particular, in models of bronchopulmonary dysplasia (BPD), exogenous MnSOD significantly alleviates pulmonary arterial hypertension levels by increasing eNOS expression (Afolayan et al., 2012). Similar to previous reports in the BPD model made from lambs, the applicatio of recombinant human CuZnSOD (rhSOD) can enhance vascular dilation ability and enhance iNO responsiveness (Steinhorn et al., 2001), further increasing the body’s oxygen level and ability to reduce oxidative stress damage (Lakshminrusimha et al., 2006). In both of vivo and vitro studies have illustrated that hydrocortisone treatment can associated with reductions in ROS levels and decreased activation of phosphodiesterase 5 (PDE5) under high-oxygen conditions (Perez et al., 2014; Perez et al., 2012), forming the theoretical foundation for utilizing hydrocortisone in BPD treatment. In the hypoxic-ischemic encephalopathy (HIE) mouse model, the downregulation of mitochondrial complex I can lead to decreased ROS levels (Kim et al., 2018b), indicating that mitochondrial complex I is a critical target for both prevention and treatment of HIE. As previously mentioned, N-acetylcysteine amide (NACA) can mitigate ROS increases triggered by LINC00938 knockout (Zhao et al., 2023). Additionally, natural compounds like myricetin and echinocystic acid have been shown to diminish oxidative stress in HIE by activating the NRF2 and PI3K/Akt/Nrf2 pathways, respectively (Chen et al., 2023; Li et al., 2023). For the rat model, intravenous administration of astragaloside, all-trans retinoic acid, or N-acetylcysteine via tail vein injection can enhance SOD and GPx activity, leading to improved outcomes in necrotizing enterocolitis (NEC) models (Aceti et al., 2018). In studies utilizing a murine NEC model, fecal microbiota transfer (FMT) has been documented to regulate oxidative stress and mitigate colitis while promoting NO production by eliminating superoxide radicals (Li et al., 2017; Ferretti et al., 2017). There exists considerable debate regarding the efficacy of vitamins E and A and trace elements in managing OS, and their role as clinical therapeutics is often constrained by biochemical and physiological factors. Some researchers are endeavoring to boost antioxidant capacity in preterm infants via supplementation of vitamins and cofactors. However, recent studies concerning BPD have shown that vitamin E and selenium did not significantly reduce the incidence of BPD (Watts et al., 1991; Darlow et al., 2000). Reports indicate that menaquinone-4 (MK-4), a vitamin K2 subtype, can activate the Sirt1-PGC-1α-TFAM signaling pathway in neonatal rat HIE models, resulting in diminished oxidative stress damage (Feng et al., 2024). There are also findings suggesting that additional vitamin E supplementation may lower the incidence and severity of retinopathy of prematurity (ROP) (Tsang et al., 2019). Vitamin E functions as a free radical scavenger, reducing lipid peroxidation during ROP’s pathological progression and contributing to retinal cell integrity maintenance (Tsang et al., 2019). Furthermore, vitamin C, as a water-soluble antioxidant, can maintain its stability by providing electrons to free radicals. It can also effectively regenerate the antioxidant form of vitamin E by reducing tocopherol free radicals (Pehlivan, 2017; Hacişevki, 2009; Arrigoni and De Tullio, 2002; Rouhier et al., 2008). As previously noted, in obese children, the levels of β-carotene and vitamin E were significantly reduced (Stenzel et al., 2018), but current evidence does not support the notion that additional supplementation of β-carotene and vitamin E can prevent obesity. A systematic review has suggested that additional supplementation of L-arginine in newborns could serve as a protective factor against NEC, although this study only included 235 infants (Mitchell et al., 2014; Table 2).

**TABLE 2:** The therapeutic prospects of neonatal oxidative stress-related diseases.

Diease	Therapy	Mechanism/ROS target	Modle	Outcome	Reference
BPD	SOD	Using liposomes to deliver therapeutic SOD to the lungs	Rat	Improved survival rate after high oxygen levels	Padmanabhan et al. (1985), Davis et al. (1985)
	Ad-MnSOD	Improved eNOS expression and function	Lamb	Relaxation response of PPHN pulmonary arteries	Afolayan et al. (2012)
	RhSOD	Increase sensitivity to S-nitrosyl-acetylpenicillamine (SNAP)	Lamb	Reduces pulmonary vascular resistance	Steinhorn et al. (2001)
	RhSOD	Relieve the increase in isoprostane levels	Lamb	Relieve pulmonary artery constriction	Lakshminrusimha et al. (2006)
	Hydrocortisone	Reduce the activity of NFκB	Fetal pulmonary artery smooth muscle cells (FPASMCs) from PPHN lambs	Reduced the level of Phosphodiesterase-5 (PDE5)	Perez et al. (2014)
	Hydrocortisone	Decreased sGC activity, Increased PDE5 activity	Lamb	increases cGMP	Perez et al. (2012)
	Melatonin	Reduce nitrate/nitrate, MDA, and increase the content of mycoxidase (MPO)	Rat	Reduce interstitial fibrosis and increase the number of alveoli in the lungs	Pan et al. (2009)
HIE	MitoSNO	Slowed down the transition of mitochondrial complex D to A	Mice	Reduced ROS generation and neuronal mortality	Kim et al. (2018b)
	NACA	Inhibiting JNK/p38 MAPK signaling pathway	peripheral blood of neonate	Inhibit oxidative stress and apoptosis of CNS	Zhao et al. (2023)
	LINC00938	Prevented the apoptosis of SH-SY5Y from OGD injury	peripheral blood of neonate	Inhibit oxidative stress and apoptosis of CNS	Zhao et al. (2023)
	Myricetin	Activate NRF2 signaling pathway	Rat	Reduce brain infarction volume, glia activation, apoptosis, and oxidative stress marker levels	Chen et al. (2023)
	Echinocystic acid	Activate the PI3K/Akt/Nrf2 signaling pathway	Mice	Reduced cerebral infarction, attenuated neuronal injury	Li et al. (2023)
	Menaquinone-4 (MK-4)	Activate Sirt1-PGC-1α-TFAM signaling pathway	Rat	Enhance mitochondrial function and exhibit protective effects against ischemia-reperfusion injury	Feng et al. (2024)
	Melatonin	Inhibition of mitochondrial cell death pathways	Mice	Reduce damage to brain nerve cells	Sinha et al. (2018)
		Reduce the production of ROS	Lamb	Reducing neurological damage	Miller et al. (2005)
		Reduce the level of activated caspase-3	Mice	Has a preventive effect on HIE at birth	Hutton et al. (2009)
NEC	All trans retinoic acid	Increase the activity of SOD and GPx	Rat	Increase the survival rate and duration of NEC rats	Aceti et al. (2018)
	N-acetylcysteine				
	astragaloside				
	Fecal microbiota transplantation (FMT)	Modulation of S-glutathionylation of eNOS (eNOS-SSG),promoted NO production	Mice	Reduced colon inflammation	Li et al. (2017)
	Epidermal growth factor (EGF)	Promoted the expression of NOS2	Human small intestine cells	Increased the survival rate of small intestinal cells	Ferretti et al. (2017)
	L-arginine	Increased synthesis of NO	Newborns with NEC	Reduced the incidence of stage II and III NEC	Mitchell et al. (2014)
ROP	Vitamin E	As a scavenger of free radicals can reduce the degree of lipid peroxidation during the pathological process	Mice	Maintain the integrity of retinal cells	Tsang et al. (2019)

In this table, we summarize some treatment methods for neonatal oxidative stress-related diseases and their specific targets, verified model information and improvement methods for disease.

It is noteworthy that melatonin shows significant promise in its antioxidant stress effects during early life. Melatonin acts as a broad-spectrum anti-apoptotic agent, antioxidant, and effective free radical scavenger (Wang, 2009; Halliwell, 1994; Tordjman et al., 2017). Research has indicated that oral administration of melatonin can substantially decrease nitrate/nitrite levels, lower MDA levels, and enhance myeloperoxidase (MPO) content, thereby alleviating pulmonary interstitial fibrosis in neonatal rats suffering from BPD and increasing alveolar counts (Pan et al., 2009). Investigations indicate that melatonin exerts neuroprotective effects in HIE mouse models (Sinha et al., 2018). Notably, Suzanne et al. confirmed through sheep models that melatonin significantly reduces ROS levels in fetal sheep, thereby minimizing neurological damage (Miller et al., 2005). Hutton et al. utilized a mouse model and found that levels of activated caspase-3 and fractions in microglia within the brains of asphyxiated offspring of mothers administered oral melatonin during pregnancy were reduced compared to the control group (Hutton et al., 2009). Additionally, Guven et al. demonstrated in their rat NEC model study that oral melatonin effectively decreased postoperative inflammatory cytokines while increasing antioxidant enzyme activity, significantly mitigating the severity of NEC (Guven et al., 2011).

## 6 Conclusion

In recent years, accumulating evidence suggests that changes related to OS play a significant role in diseases associated with the prenatal period. In fetal development-related conditions, such as intrauterine growth restriction (IUGR), critical indicators of oxidative stress, including 8-oxodG and D-ROM, are markedly elevated. Similarly, in fetuses affected by twin-to-twin transfusion syndrome, metabolites indicative of heightened oxidative stress, including MDA and TBARS, demonstrated significant increases. *In vitro* studies have shown that an increase in eNOS and ROS levels considerably impacts the integrity of fetal cardiac development. In recent years, the latest research has shown that Histone deacetylases (HDACs) play an exciting role in early cardiac injury repair (Zhu et al., 2024a; Zuo et al., 2025).

The elevation of 8-isoprostane, MDA, and GSH is also related to the risk of gestational diabetes. Interestingly, the levels of SOD1 and SOD in the placenta of mothers with gestational diabetes exhibit inconsistencies.

There are two important mechanisms underlying oxidative stress events in women with preeclampsia, one being the disruption of the NO/NOS system. The main manifestation is a decrease in nitrogen oxide levels and an increase in arginase levels (Lowe, 2000; Dai et al., 2013). Another important mechanism is mediated by an increase in ROS. Studies have shown that women with early-onset preeclampsia have a higher rate of superoxide production compared to women with late onset preeclampsia (Raijmakers et al., 2004).

Among neonatal diseases, notable elevations in c-Abl, NOX1, and GSSG/GSH levels have been observed in infants with BPD. NEC-related conditions show significantly increased levels of advanced oxidation protein products (AOPP), H_2_O_2_, and total oxidant status (TOS). The level of ROS is substantially elevated in HIE-related diseases, and it is worth noting that the PI3K/Akt/Nrf2 signaling pathway is significantly inhibited.

Regarding childhood nutrition and endocrine system diseases, analysis of childhood obesity revealed increased levels of TOS, TBARS, and isoprostane. Interestingly, total antioxidant status (TAS) also showed a corresponding increase, while levels of retinol, β-carotene, and vitamin E exhibited a significant decline. In studies focused on children with diabetes, a notable increase in total Cu/Zn values for those with T1DM was observed, accompanied by heightened MDA levels, with TAS levels showing a decrease. In children diagnosed with T2DM, mitochondrial oxidative stress levels were elevated while G6PD levels decreased. Regarding GHD among children, SIRT1 levels were significantly diminished in both animal models and human cells. In the latest research, shown that the oxidative balance score (OBS) is inversely proportional to the prognosis of children with metabolic syndrome, including those with obesity. Therefore, it is crucial to utilize OBS to adhere to an antioxidant diet and lifestyle (Zhu et al., 2024b).

To date, treatment strategies for perinatal oxidative stress-related diseases primarily focus on neonatal conditions. Traditional studies on cognitive oxidative stress management have concentrated on enzymes such as CuZnSOD, MnSOD, ecSOD, and GSH. Hydrocortisone use for the treatment of BPD has gained widespread acceptance in clinical practice, while melatonin also offers potential therapeutic benefits for BPD and HIE. Compounds such as NACA, LINC00938, myricetin, echinocystic acid, and menaquinone-4 (MK-4) display varied therapeutic efficacy in HIE treatment. In managing NEC, administering certain amino acids, such as N-acetylcysteine, all-trans retinoic acid, and L-arginine through various routes, could yield distinct advantages for long-term disease prognosis. Furthermore, approaches like astragaloside, fecal microbiota transfer (FMT), and epidermal growth factor (EGF) have also contributed to improving NEC management to varying extents. Vitamin E has demonstrated significant effectiveness in enhancing the prognosis of infants diagnosed with ROP.

## References

[B1] Aceti A. Beghetti I. Martini S. Faldella G. Corvaglia L. (2018). Oxidative stress and necrotizing enterocolitis: pathogenetic mechanisms, opportunities for intervention, and role of human milk. Oxid. Med. Cell Longev. 2018, 7397659. 10.1155/2018/7397659 30057683 PMC6051049

[B2] ACOG Practice Bulletin (2018). ACOG practice bulletin no. 190: gestational diabetes mellitus. Obstet. Gynecol. 131, e49–e64. 10.1097/AOG.0000000000002501 29370047

[B3] Afolayan A. J. Eis A. Teng R. J. Bakhutashvili I. Kaul S. Davis J. M. (2012). Decreases in manganese superoxide dismutase expression and activity contribute to oxidative stress in persistent pulmonary hypertension of the newborn. Am. J. Physiol. Lung Cell Mol. Physiol. 303, L870–L879. 10.1152/ajplung.00098.2012 22962015 PMC3517675

[B4] Alajbegovic-Halimic J. Zvizdic D. Alimanovic-Halilovic E. Dodik I. Duvnjak S. (2015). Risk factors for retinopathy of prematurity in premature born children. Med. Arch. 69, 409–413. 10.5455/medarh.2015.69.409-413 26843736 PMC4720470

[B5] Al-Shabrawey M. Bartoli M. El-Remessy A. B. Platt D. H. Matragoon S. Behzadian M. A. (2005). Inhibition of NAD(P)H oxidase activity blocks vascular endothelial growth factor overexpression and neovascularization during ischemic retinopathy. Am. J. Pathol. 167, 599–607. 10.1016/S0002-9440(10)63001-5 16049343 PMC1603550

[B6] Alu S. N. Los E. A. Ford G. A. Stone W. L. (2022). Oxidative stress in type 2 diabetes: the case for future pediatric redoxomics studies. Antioxidants (Basel) 11, 1336. 10.3390/antiox11071336 35883827 PMC9312244

[B7] Arrigoni O. De Tullio M. C. (2002). Ascorbic acid: much more than just an antioxidant. Biochim. Biophys. Acta 1569, 1–9. 10.1016/s0304-4165(01)00235-5 11853951

[B8] Asano K. Cantalupo A. Sedes L. Ramirez F. (2022). Pathophysiology and therapeutics of thoracic aortic aneurysm in Marfan syndrome. Biomolecules 12, 128. 10.3390/biom12010128 35053276 PMC8773516

[B9] Ashina M. Kido T. Kyono Y. Yoshida A. Suga S. Nakasone R. (2021). Correlation between severity of fetal growth restriction and oxidative stress in severe small-for-gestational-age infants. Int. J. Environ. Res. Public Health 18, 10726. 10.3390/ijerph182010726 34682470 PMC8535480

[B10] Asikainen T. M. White C. W. (2004). Pulmonary antioxidant defenses in the preterm newborn with respiratory distress and bronchopulmonary dysplasia in evolution: implications for antioxidant therapy. Antioxid. Redox Signal 6, 155–167. 10.1089/152308604771978462 14713347

[B11] Aydemir C. Dilli D. Uras N. Ulu H. O. Oguz S. S. Erdeve O. (2011). Total oxidant status and oxidative stress are increased in infants with necrotizing enterocolitis. J. Pediatr. Surg. 46, 2096–2100. 10.1016/j.jpedsurg.2011.06.032 22075338

[B12] Baregamian N. Song J. Papaconstantinou J. Hawkins H. K. Evers B. M. Chung D. H. (2011). Intestinal mitochondrial apoptotic signaling is activated during oxidative stress. Pediatr. Surg. Int. 27, 871–877. 10.1007/s00383-011-2880-x 21400030 PMC3668660

[B13] Beauchamp M. H. Sennlaub F. Speranza G. Gobeil F. Jr. Checchin D. Kermorvant-Duchemin E. (2004). Redox-dependent effects of nitric oxide on microvascular integrity in oxygen-induced retinopathy. Free Radic. Biol. Med. 37, 1885–1894. 10.1016/j.freeradbiomed.2004.09.008 15528047

[B14] Benjamin E. J. Virani S. S. Callaway C. W. Chamberlain A. M. Chang A. R. Cheng S. (2018). Heart disease and stroke Statistics-2018 update: a report from the American heart association. Circulation 137, e67–e492. 10.1161/CIR.0000000000000558 29386200

[B15] Berkelhamer S. K. Farrow K. N. (2014). Developmental regulation of antioxidant enzymes and their impact on neonatal lung disease. Antioxid. Redox Signal 21, 1837–1848. 10.1089/ars.2013.5515 24295375 PMC4203145

[B16] Bloch W. Fleischmann B. K. Lorke D. E. Andressen C. Hops B. Hescheler J. (1999). Nitric oxide synthase expression and role during cardiomyogenesis. Cardiovasc Res. 43, 675–684. 10.1016/s0008-6363(99)00160-1 10690339

[B17] Bonnefont X. Lacampagne A. Sanchez-Hormigo A. Fino E. Creff A. Mathieu M. N. (2005). Revealing the large-scale network organization of growth hormone-secreting cells. Proc. Natl. Acad. Sci. U. S. A. 102, 16880–16885. 10.1073/pnas.0508202102 16272219 PMC1277257

[B18] Brooks S. E. Gu X. Samuel S. Marcus D. M. Bartoli M. Huang P. L. (2001). Reduced severity of oxygen-induced retinopathy in eNOS-deficient mice. Invest Ophthalmol. Vis. Sci. 42, 222–228. 11133872

[B19] Budbazar E. Sulser Ponce De Leon S. Tsukahara Y. Liu H. Huangfu Y. Wang Y. (2023). Redox dysregulation of vascular smooth muscle Sirtuin-1 in thoracic aortic aneurysm in Marfan syndrome. Arterioscler. Thromb. Vasc. Biol. 43, e339–e357. 10.1161/ATVBAHA.123.319145 37288573 PMC10524979

[B20] Budinger G. R. Mutlu G. M. Urich D. Soberanes S. Buccellato L. J. Hawkins K. (2011). Epithelial cell death is an important contributor to oxidant-mediated acute lung injury. Am. J. Respir. Crit. Care Med. 183, 1043–1054. 10.1164/rccm.201002-0181OC 20959557 PMC3086743

[B21] Buonocore G. Perrone S. Longini M. Vezzosi P. Marzocchi B. Paffetti P. (2002). Oxidative stress in preterm neonates at birth and on the seventh day of life. Pediatr. Res. 52, 46–49. 10.1203/00006450-200207000-00010 12084846

[B22] Cao Y. Jiang X. Ma H. Wang Y. Xue P. Liu Y. (2016). SIRT1 and insulin resistance. J. Diabetes Complicat. 30, 178–183. 10.1016/j.jdiacomp.2015.08.022 26422395

[B23] Carnesecchi S. Deffert C. Pagano A. Garrido-Urbani S. Metrailler-Ruchonnet I. Schappi M. (2009). NADPH oxidase-1 plays a crucial role in hyperoxia-induced acute lung injury in mice. Am. J. Respir. Crit. Care Med. 180, 972–981. 10.1164/rccm.200902-0296OC 19661248 PMC2778156

[B24] Chang L. Y. Subramaniam M. Yoder B. A. Day B. J. Ellison M. C. Sunday M. E. (2003b). A catalytic antioxidant attenuates alveolar structural remodeling in bronchopulmonary dysplasia. Am. J. Respir. Crit. Care Med. 167, 57–64. 10.1164/rccm.200203-232OC 12502477

[B25] Chang T. I. Horal M. Jain S. K. Wang F. Patel R. Loeken M. R. (2003a). Oxidant regulation of gene expression and neural tube development: insights gained from diabetic pregnancy on molecular causes of neural tube defects. Diabetologia 46, 538–545. 10.1007/s00125-003-1063-2 12739027

[B26] Chang Y. L. Wang C. N. Wei P. C. Peng H. H. Chao A. S. Chang S. D. (2013). Mitochondrial activation in the growth-restricted fetus of monochorionic twins. Fertil. Steril. 100, 241-246 e241–246. 10.1016/j.fertnstert.2013.03.003 23557760

[B27] Chang Y. S. Ahn S. Y. Sung S. Park W. S. (2017). Stem cell therapy for neonatal disorders: prospects and challenges. Yonsei Med. J. 58, 266–271. 10.3349/ymj.2017.58.2.266 28120555 PMC5290004

[B28] Chaudhari L. Tandon O. P. Vaney N. Agarwal N. (2003). Lipid peroxidation and antioxidant enzymes in gestational diabetics. Indian J. Physiol. Pharmacol. 47, 441–446. 15266957

[B29] Chen C. Zhou M. Ge Y. Wang X. (2020). SIRT1 and aging related signaling pathways. Mech. Ageing Dev. 187, 111215. 10.1016/j.mad.2020.111215 32084459

[B30] Chen T. Hu Y. Lu L. Zhao Q. Tao X. Ding B. (2023). Myricetin attenuates hypoxic-ischemic brain damage in neonatal rats via NRF2 signaling pathway. Front. Pharmacol. 14, 1134464. 10.3389/fphar.2023.1134464 36969871 PMC10031108

[B31] Chen X. Li J. Kang R. Klionsky D. J. Tang D. (2021). Ferroptosis: machinery and regulation. Autophagy 17, 2054–2081. 10.1080/15548627.2020.1810918 32804006 PMC8496712

[B32] Cleves M. A. Ghaffar S. Zhao W. Mosley B. S. Hobbs C. A. (2003). First-year survival of infants born with congenital heart defects in Arkansas (1993-1998): a survival analysis using registry data. Birth Defects Res. A Clin. Mol. Teratol. 67, 662–668. 10.1002/bdra.10119 14703791

[B33] Consortium R. (2018). Impact of insulin and metformin Versus metformin alone on beta-cell function in youth with impaired glucose tolerance or recently diagnosed type 2 diabetes. Diabetes Care 41, 1717–1725. 10.2337/dc18-0787 29941500 PMC6054504

[B34] Correia-Costa L. Sousa T. Morato M. Cosme D. Afonso J. Areias J. C. (2016). Oxidative stress and nitric oxide are increased in obese children and correlate with cardiometabolic risk and renal function. Br. J. Nutr. 116, 805–815. 10.1017/S0007114516002804 27480380

[B35] Coughlan M. T. Vervaart P. P. Permezel M. Georgiou H. M. Rice G. E. (2004). Altered placental oxidative stress status in gestational diabetes mellitus. Placenta 25, 78–84. 10.1016/S0143-4004(03)00183-8 15013642

[B36] Courtney S. E. Durand D. J. Asselin J. M. Hudak M. L. Aschner J. L. Shoemaker C. T. (2002). Neonatal ventilation study G: **High-frequency oscillatory ventilation versus conventional mechanical ventilation for very-low-birth-weight infants** . N. Engl. J. Med. 347, 643–652. 10.1056/NEJMoa012750 12200551

[B37] Dai B. Liu T. Zhang B. Zhang X. Wang Z. (2013). The polymorphism for endothelial nitric oxide synthase gene, the level of nitric oxide and the risk for pre-eclampsia: a meta-analysis. Gene 519, 187–193. 10.1016/j.gene.2013.01.004 23375994

[B38] Darlow B. A. Winterbourn C. C. Inder T. E. Graham P. J. Harding J. E. Weston P. J. (2000). The effect of selenium supplementation on outcome in very low birth weight infants: a randomized controlled trial. The New Zealand neonatal study group. J. Pediatr. 136, 473–480. 10.1016/s0022-3476(00)90010-6 10753245

[B39] Datta A. Kim G. A. Taylor J. M. Gugino S. F. Farrow K. N. Schumacker P. T. (2015). Mouse lung development and NOX1 induction during hyperoxia are developmentally regulated and mitochondrial ROS dependent. Am. J. Physiol. Lung Cell Mol. Physiol. 309, L369–L377. 10.1152/ajplung.00176.2014 26092998 PMC4587628

[B40] Davis J. M. Rosenfeld W. N. Sanders R. J. Gonenne A. (1985). Prophylactic effects of recombinant human superoxide dismutase in neonatal lung injury. J. Appl. Physiol. 74 (74), 2234–2241. 10.1152/jappl.1993.74.5.2234 8335553

[B41] Dede H. Takmaz O. Ozbasli E. Dede S. Gungor M. (2017). Higher level of oxidative stress markers in small for gestational age newborns delivered by cesarean section at term. Fetal Pediatr. Pathol. 36, 232–239. 10.1080/15513815.2017.1303860 28368675

[B42] Denbow M. L. Cox P. Taylor M. Hammal D. M. Fisk N. M. (2000). Placental angioarchitecture in monochorionic twin pregnancies: relationship to fetal growth, fetofetal transfusion syndrome, and pregnancy outcome. Am. J. Obstet. Gynecol. 182, 417–426. 10.1016/s0002-9378(00)70233-x 10694346

[B43] DeRuiter M. C. Poelmann R. E. VanderPlas-de Vries I. Mentink M. M. Gittenberger-de Groot A. C. (1992). The development of the myocardium and endocardium in mouse embryos. Fusion of two heart tubes? Anat. Embryol. Berl. 185, 461–473. 10.1007/BF00174084 1567022

[B44] Dixon S. J. Lemberg K. M. Lamprecht M. R. Skouta R. Zaitsev E. M. Gleason C. E. (2012). Ferroptosis: an iron-dependent form of nonapoptotic cell death. Cell 149, 1060–1072. 10.1016/j.cell.2012.03.042 22632970 PMC3367386

[B45] Dong D. Zhang Y. Reece E. A. Wang L. Harman C. R. Yang P. (2016). microRNA expression profiling and functional annotation analysis of their targets modulated by oxidative stress during embryonic heart development in diabetic mice. Reprod. Toxicol. 65, 365–374. 10.1016/j.reprotox.2016.09.007 27629361 PMC5288404

[B46] ElSayed N. A. Aleppo G. Aroda V. R. Bannuru R. R. Brown F. M. Bruemmer D. (2023). 2. Classification and diagnosis of diabetes: standards of care in Diabetes-2023. Diabetes Care 46, S19–S40. 10.2337/dc23-S002 36507649 PMC9810477

[B47] Feng Q. Song W. Lu X. Hamilton J. A. Lei M. Peng T. (2002). Development of heart failure and congenital septal defects in mice lacking endothelial nitric oxide synthase. Circulation 106, 873–879. 10.1161/01.cir.0000024114.82981.ea 12176963

[B48] Feng X. Zheng Y. Mao N. Shen M. Chu L. Fang Y. (2024). Menaquinone-4 alleviates hypoxic-ischemic brain damage in neonatal rats by reducing mitochondrial dysfunction via Sirt1-PGC-1α-TFAM signaling pathway. Int. Immunopharmacol. 134, 112257. 10.1016/j.intimp.2024.112257 38759366

[B49] Ferretti E. Tremblay E. Thibault M. P. Grynspan D. Burghardt K. M. Bettolli M. (2017). The nitric oxide synthase 2 pathway is targeted by both pro- and anti-inflammatory treatments in the immature human intestine. Nitric Oxide 66, 53–61. 10.1016/j.niox.2017.03.003 28315470

[B50] Formanowicz D. Malinska A. Nowicki M. Kowalska K. Gruca-Stryjak K. Breborowicz G. (2019). Preeclampsia with intrauterine growth restriction generates morphological changes in endothelial cells associated with mitochondrial Swelling-An *in vitro* study. J. Clin. Med. 8, 1994. 10.3390/jcm8111994 31731752 PMC6912746

[B51] Ge T. Yang J. Zhou S. Wang Y. Li Y. Tong X. (2020). The role of the pentose phosphate pathway in diabetes and cancer. Front. Endocrinol. (Lausanne) 11, 365. 10.3389/fendo.2020.00365 32582032 PMC7296058

[B52] Geca T. Stupak A. Nawrot R. Gozdzicka-Jozefiak A. Kwasniewska A. Kwasniewski W. (2022). Placental proteome in late-onset of fetal growth restriction. Mol. Med. Rep. 26, 356. 10.3892/mmr.2022.12872 36263610 PMC9608316

[B53] Gilboa S. M. Devine O. J. Kucik J. E. Oster M. E. Riehle-Colarusso T. Nembhard W. N. (2016). Congenital heart defects in the United States: estimating the magnitude of the affected population in 2010. Circulation 134, 101–109. 10.1161/CIRCULATIONAHA.115.019307 27382105 PMC4942347

[B54] Grabia M. Socha K. Soroczynska J. Bossowski A. Markiewicz-Zukowska R. (2023). Determinants related to oxidative stress parameters in pediatric patients with type 1 diabetes mellitus. Nutrients 15, 2084. 10.3390/nu15092084 37432230 PMC10180949

[B55] Gu X. El-Remessy A. B. Brooks S. E. Al-Shabrawey M. Tsai N. T. Caldwell R. B. (2003). Hyperoxia induces retinal vascular endothelial cell apoptosis through formation of peroxynitrite. Am. J. Physiol. Cell Physiol. 285, C546–C554. 10.1152/ajpcell.00424.2002 12736139

[B56] Gupta P. Narang M. Banerjee B. D. Basu S. (2004). Oxidative stress in term small for gestational age neonates born to undernourished mothers: a case control study. BMC Pediatr. 4, 14. 10.1186/1471-2431-4-14 15260886 PMC487903

[B57] Gur S. Turk G. Demirci E. Yuce A. Sonmez M. Ozer S. (2011). Effect of pregnancy and foetal number on diameter of corpus luteum, maternal progesterone concentration and oxidant/antioxidant balance in ewes. Reprod. Domest. Anim. 46, 289–295. 10.1111/j.1439-0531.2010.01660.x 20565696

[B58] Guven A. Uysal B. Gundogdu G. Oztas E. Ozturk H. Korkmaz A. (2011). Melatonin ameliorates necrotizing enterocolitis in a neonatal rat model. J. Pediatr. Surg. 46, 2101–2107. 10.1016/j.jpedsurg.2011.06.040 22075339

[B59] Hacişevki A. (2009). An overview of ascorbic acid biochemistry. Ank. Univ. Eczacilik Fak. Derg. 38, 233–255. 10.1501/eczfak_0000000528

[B60] Hackam D. Caplan M. (2018). Necrotizing enterocolitis: pathophysiology from a historical context. Semin. Pediatr. Surg. 27, 11–18. 10.1053/j.sempedsurg.2017.11.003 29275810 PMC6207945

[B61] Haigis M. C. Sinclair D. A. (2010). Mammalian sirtuins: biological insights and disease relevance. Annu. Rev. Pathol. 5, 253–295. 10.1146/annurev.pathol.4.110807.092250 20078221 PMC2866163

[B62] Halliwell B. (1994). Free radicals, antioxidants, and human disease: curiosity, cause, or consequence? Lancet 344, 721–724. 10.1016/s0140-6736(94)92211-x 7915779

[B63] Handy D. E. Lubos E. Yang Y. Galbraith J. D. Kelly N. Zhang Y. Y. (2009). Glutathione peroxidase-1 regulates mitochondrial function to modulate redox-dependent cellular responses. J. Biol. Chem. 284, 11913–11921. 10.1074/jbc.M900392200 19254950 PMC2673260

[B64] Hellstrom A. Perruzzi C. Ju M. Engstrom E. Hard A. L. Liu J. L. (2001). Low IGF-I suppresses VEGF-survival signaling in retinal endothelial cells: direct correlation with clinical retinopathy of prematurity. Proc. Natl. Acad. Sci. U. S. A. 98, 5804–5808. 10.1073/pnas.101113998 11331770 PMC33294

[B65] Heymann A. D. Cohen Y. Chodick G. (2012). Glucose-6-phosphate dehydrogenase deficiency and type 2 diabetes. Diabetes Care 35, e58. 10.2337/dc11-2527 22826451 PMC3402279

[B66] Horbar J. D. Badger G. J. Carpenter J. H. Fanaroff A. A. Kilpatrick S. LaCorte M. (2002). Members of the Vermont Oxford N: **Tretds in mortality and morbidity for very low birth weight infants, 1991-1999** . Pediatrics 110, 143–151. 10.1542/peds.110.1.143 12093960

[B67] Hutton L. C. Abbass M. Dickinson H. Ireland Z. Walker D. W. (2009). Neuroprotective properties of melatonin in a model of birth asphyxia in the spiny mouse (Acomys cahirinus). Dev. Neurosci. 31, 437–451. 10.1159/000232562 19684403

[B68] Ighodaro O. M. Akinloye O. A. (2019). First line defence antioxidants-superoxide dismutase (SOD), catalase (CAT) and glutathione peroxidase (GPX): their fundamental role in the entire antioxidant defence grid. Alexandria J. Med. 54, 287–293. 10.1016/j.ajme.2017.09.001

[B69] Ilizarov A. M. Koo H. C. Kazzaz J. A. Mantell L. L. Li Y. Bhapat R. (2001). Overexpression of manganese superoxide dismutase protects lung epithelial cells against oxidant injury. Am. J. Respir. Cell Mol. Biol. 24, 436–441. 10.1165/ajrcmb.24.4.4240 11306437

[B70] Imperatore G. Boyle J. P. Thompson T. J. Case D. Dabelea D. Hamman R. F. (2012). Projections of type 1 and type 2 diabetes burden in the U.S. population aged <20 years through 2050: dynamic modeling of incidence, mortality, and population growth. Diabetes Care 35, 2515–2520. 10.2337/dc12-0669 23173134 PMC3507562

[B71] Jantsch L. B. de Lucca L. Dorneles B. N. Konopka C. K. Goncalves T. L. (2020). Evaluation of oxidative stress and delta-aminolevulinate dehydratase activity in twin pregnancies. J. Matern. Fetal Neonatal Med. 33, 3071–3076. 10.1080/14767058.2019.1568980 30688119

[B72] Jomova K. Makova M. Alomar S. Y. Alwasel S. H. Nepovimova E. Kuca K. (2022). Essential metals in health and disease. Chem. Biol. Interact. 367, 110173. 10.1016/j.cbi.2022.110173 36152810

[B73] Kilic E. Ozer O. F. Erek Toprak A. Erman H. Torun E. Kesgin Ayhan S. (2016). Oxidative stress status in childhood obesity: a potential risk predictor. Med. Sci. Monit. 22, 3673–3679. 10.12659/msm.897965 27733746 PMC5066503

[B74] Kim M. Stepanova A. Niatsetskaya Z. Sosunov S. Arndt S. Murphy M. P. (2018b). Attenuation of oxidative damage by targeting mitochondrial complex I in neonatal hypoxic-ischemic brain injury. Free Radic. Biol. Med. 124, 517–524. 10.1016/j.freeradbiomed.2018.06.040 30037775 PMC6389362

[B75] Kim S. J. Port A. D. Swan R. Campbell J. P. Chan R. V. P. Chiang M. F. (2018a). Retinopathy of prematurity: a review of risk factors and their clinical significance. Surv. Ophthalmol. 63, 618–637. 10.1016/j.survophthal.2018.04.002 29679617 PMC6089661

[B76] Kinalski M. Sledziewski A. Telejko B. Kowalska I. Kretowski A. Zarzycki W. (2001). Lipid peroxidation, antioxidant defence and acid-base status in cord blood at birth: the influence of diabetes. Horm. Metab. Res. 33, 227–231. 10.1055/s-2001-14953 11383927

[B77] Knowles R. G. Moncada S. (1994). Nitric oxide synthases in mammals. Biochem. J. 298 (Pt 2), 249–258. 10.1042/bj2980249 7510950 PMC1137932

[B78] Koo H. C. Davis J. M. Li Y. Hatzis D. Opsimos H. Pollack S. (2005). Effects of transgene expression of superoxide dismutase and glutathione peroxidase on pulmonary epithelial cell growth in hyperoxia. Am. J. Physiol. Lung Cell Mol. Physiol. 288, L718–L726. 10.1152/ajplung.00456.2003 15579623

[B79] Lakshminrusimha S. Russell J. A. Wedgwood S. Gugino S. F. Kazzaz J. A. Davis J. M. (2006). Superoxide dismutase improves oxygenation and reduces oxidation in neonatal pulmonary hypertension. Am. J. Respir. Crit. Care Med. 174, 1370–1377. 10.1164/rccm.200605-676OC 17008638 PMC2111046

[B80] Lappas M. Permezel M. Rice G. E. (2004). Release of proinflammatory cytokines and 8-isoprostane from placenta, adipose tissue, and skeletal muscle from normal pregnant women and women with gestational diabetes mellitus. J. Clin. Endocrinol. Metab. 89, 5627–5633. 10.1210/jc.2003-032097 15531521

[B81] Lechuga-Sancho A. M. Gallego-Andujar D. Ruiz-Ocana P. Visiedo F. M. Saez-Benito A. Schwarz M. (2018). Obesity induced alterations in redox homeostasis and oxidative stress are present from an early age. PLoS One 13, e0191547. 10.1371/journal.pone.0191547 29370267 PMC5784965

[B82] Lee H. C. Yin P. H. Lu C. Y. Chi C. W. Wei Y. H. (2000). Increase of mitochondria and mitochondrial DNA in response to oxidative stress in human cells. Biochem. J. 348 (Pt 2), 425–432. 10.1042/bj3480425 10816438 PMC1221082

[B83] Lee S. H. Lee J. H. Lee H. Y. Min K. J. (2019). Sirtuin signaling in cellular senescence and aging. BMB Rep. 52, 24–34. 10.5483/BMBRep.2019.52.1.290 30526767 PMC6386230

[B84] Leo F. Rossodivita A. N. Segni C. D. Raimondo S. Canichella S. Silvestrini A. (2016). Frailty of obese children: evaluation of plasma antioxidant capacity in pediatric obesity. Exp. Clin. Endocrinol. Diabetes 124, 481–486. 10.1055/s-0042-105280 27169687

[B85] Li X. Li X. Shang Q. Gao Z. Hao F. Guo H. (2017). Fecal microbiota transplantation (FMT) could reverse the severity of experimental necrotizing enterocolitis (NEC) via oxidative stress modulation. Free Radic. Biol. Med. 108, 32–43. 10.1016/j.freeradbiomed.2017.03.011 28323128

[B86] Li Y. Chen L. Zheng D. Liu J. X. Liu C. Qi S. H. (2023). Echinocystic acid alleviated hypoxic-ischemic brain damage in neonatal mice by activating the PI3K/Akt/Nrf2 signaling pathway. Front. Pharmacol. 14, 1103265. 10.3389/fphar.2023.1103265 36843928 PMC9947717

[B87] Li Y. Tao Y. Xu J. He Y. Zhang W. Jiang Z. (2021). Hyperoxia provokes Time- and dose-dependent gut injury and endotoxemia and alters gut microbiome and transcriptome in mice. Front. Med. (Lausanne) 8, 732039. 10.3389/fmed.2021.732039 34869425 PMC8635731

[B88] Liu X. Li T. Liu Y. Sun S. Liu D. (2021). Nuclear factor erythroid 2-related factor 2 potentiates the generation of inflammatory cytokines by intestinal epithelial cells during hyperoxia by inducing the expression of interleukin 17D. Toxicology 457, 152820. 10.1016/j.tox.2021.152820 34023435

[B89] Liu Y. Feng Q. (2012). NOing the heart: role of nitric oxide synthase-3 in heart development. Differentiation 84, 54–61. 10.1016/j.diff.2012.04.004 22579300

[B90] Lowe D. T. (2000). Nitric oxide dysfunction in the pathophysiology of preeclampsia. Nitric Oxide 4, 441–458. 10.1006/niox.2000.0296 10944429

[B91] Luyt K. Jary S. L. Lea C. L. Young G. J. Odd D. E. Miller H. E. (2020). Drainage, irrigation and fibrinolytic therapy (DRIFT) for posthaemorrhagic ventricular dilatation: 10-year follow-up of a randomised controlled trial. Arch. Dis. Child. Fetal Neonatal Ed. 105, 466–473. 10.1136/archdischild-2019-318231 32623370 PMC7547901

[B92] Madazli R. Tuten A. Calay Z. Uzun H. Uludag S. Ocak V. (2008). The incidence of placental abnormalities, maternal and cord plasma malondialdehyde and vascular endothelial growth factor levels in women with gestational diabetes mellitus and nondiabetic controls. Gynecol. Obstet. Invest 65, 227–232. 10.1159/000113045 18196904

[B93] Mahmoud A. A. Nor El-Din A. K. (2013). Glucose-6-Phosphate dehydrogenase activity and protein oxidative modification in patients with type 2 diabetes mellitus. J. Biomark. 2013, 430813. 10.1155/2013/430813 26317017 PMC4437381

[B94] Manoharan B. Bobby Z. Dorairajan G. Jacob S. E. Gladwin V. Vinayagam V. (2019). Increased placental expressions of nuclear factor erythroid 2-related factor 2 and antioxidant enzymes in gestational diabetes: protective mechanisms against the placental oxidative stress? Eur. J. Obstet. Gynecol. Reprod. Biol. 238, 78–85. 10.1016/j.ejogrb.2019.05.016 31121342

[B95] Marino G. Niso-Santano M. Baehrecke E. H. Kroemer G. (2014). Self-consumption: the interplay of autophagy and apoptosis. Nat. Rev. Mol. Cell Biol. 15, 81–94. 10.1038/nrm3735 24401948 PMC3970201

[B96] Matough F. A. Budin S. B. Hamid Z. A. Alwahaibi N. Mohamed J. (2012). The role of oxidative stress and antioxidants in diabetic complications. Sultan Qaboos Univ. Med. J. 12, 5–18. 10.12816/0003082 22375253 PMC3286717

[B97] Mayer-Davis E. J. Kahkoska A. R. Jefferies C. Dabelea D. Balde N. Gong C. X. (2018). ISPAD clinical practice consensus guidelines 2018: definition, epidemiology, and classification of diabetes in children and adolescents. Pediatr. Diabetes 19 (Suppl. 27), 7–19. 10.1111/pedi.12773 PMC7521365 30226024

[B98] McElroy S. J. Underwood M. A. Sherman M. P. (2013). Paneth cells and necrotizing enterocolitis: a novel hypothesis for disease pathogenesis. Neonatology 103, 10–20. 10.1159/000342340 23006982 PMC3609425

[B99] Miller S. L. Yan E. B. Castillo-Melendez M. Jenkin G. Walker D. W. (2005). Melatonin provides neuroprotection in the late-gestation fetal sheep brain in response to umbilical cord occlusion. Dev. Neurosci. 27, 200–210. 10.1159/000085993 16046855

[B100] Mitchell K. Lyttle A. Amin H. Shaireen H. Robertson H. L. Lodha A. K. (2014). Arginine supplementation in prevention of necrotizing enterocolitis in the premature infant: an updated systematic review. BMC Pediatr. 14, 226. 10.1186/1471-2431-14-226 25205007 PMC4166475

[B101] Moazzen H. Lu X. Liu M. Feng Q. (2015). Pregestational diabetes induces fetal coronary artery malformation via reactive oxygen species signaling. Diabetes 64, 1431–1443. 10.2337/db14-0190 25422104

[B102] Moazzen H. Lu X. Ma N. L. Velenosi T. J. Urquhart B. L. Wisse L. J. (2014). N-Acetylcysteine prevents congenital heart defects induced by pregestational diabetes. Cardiovasc Diabetol. 13, 46. 10.1186/1475-2840-13-46 24533448 PMC3942143

[B103] Nabhan A. N. Brownfield D. G. Harbury P. B. Krasnow M. A. Desai T. J. (2018). Single-cell Wnt signaling niches maintain stemness of alveolar type 2 cells. Science 359, 1118–1123. 10.1126/science.aam6603 29420258 PMC5997265

[B104] Nardiello C. Mizikova I. Silva D. M. Ruiz-Camp J. Mayer K. Vadasz I. (2017). Standardisation of oxygen exposure in the development of mouse models for bronchopulmonary dysplasia. Dis. Model Mech. 10, 185–196. 10.1242/dmm.027086 28067624 PMC5312005

[B105] Navarro A. Alonso A. Garrido P. Gonzalez C. Gonzalez Del Rey C. Ordonez C. (2010). Increase in placental apolipoprotein D as an adaptation to human gestational diabetes. Placenta 31, 25–31. 10.1016/j.placenta.2009.11.002 19944460 PMC7124627

[B106] Neu J. Walker W. A. (2011). Necrotizing enterocolitis. N. Engl. J. Med. 364, 255–264. 10.1056/NEJMra1005408 21247316 PMC3628622

[B107] Nordlund B. James A. Ebersjö C. Hedlin G. Broström E. B. (2017). Differences and similarities between bronchopulmonary dysplasia and asthma in schoolchildren. Pediatr. Pulmonol. 52, 1179–1186. 10.1002/ppul.23741 28636794

[B108] Northway W. H. Jr. (1967). Rosan RC, porter DY: Pulponary disease following respirator therapy of hyaline-membrane disease. Bronchopulmonary dysplasia. N. Engl. J. Med. 276, 357–368. 10.1056/NEJM196702162760701 5334613

[B109] Padmanabhan R. V. Gudapaty R. Liener I. E. Schwartz B. A. Hoidal J. R. (1985). Protection against pulmonary oxygen toxicity in rats by the intratracheal administration of liposome-encapsulated superoxide dismutase or catalase. Am. Rev. Respir. Dis. 132, 164–167. 10.1164/arrd.1985.132.1.164 4014861

[B110] Pan L. Fu J. H. Xue X. D. Xu W. Zhou P. Wei B. (2009). Melatonin protects against oxidative damage in a neonatal rat model of bronchopulmonary dysplasia. World J. Pediatr. 5, 216–221. 10.1007/s12519-009-0041-2 19693467

[B111] Pehlivan F. E. (2017). “Vitamin C: an antioxidant agent,” in Vitamin C.

[B112] Perez M. Lakshminrusimha S. Wedgwood S. Czech L. Gugino S. F. Russell J. A. (2012). Hydrocortisone normalizes oxygenation and cGMP regulation in lambs with persistent pulmonary hypertension of the newborn. Am. J. Physiol. Lung Cell Mol. Physiol. 302, L595–L603. 10.1152/ajplung.00145.2011 22198909 PMC3311533

[B113] Perez M. Wedgwood S. Lakshminrusimha S. Farrow K. N. Steinhorn R. H. (2014). Hydrocortisone normalizes phosphodiesterase-5 activity in pulmonary artery smooth muscle cells from lambs with persistent pulmonary hypertension of the newborn. Pulm. Circ. 4, 71–81. 10.1086/674903 25006423 PMC4070767

[B114] Perrone S. Tataranno M. L. Negro S. Longini M. Marzocchi B. Proietti F. (2010). Early identification of the risk for free radical-related diseases in preterm newborns. Early Hum. Dev. 86, 241–244. 10.1016/j.earlhumdev.2010.03.008 20466493

[B115] Phillippi J. A. Eskay M. A. Kubala A. A. Pitt B. R. Gleason T. G. (2010). Altered oxidative stress responses and increased type I collagen expression in bicuspid aortic valve patients. Ann. Thorac. Surg. 90, 1893–1898. 10.1016/j.athoracsur.2010.07.069 21095332 PMC8172083

[B116] Pierpont M. E. Basson C. T. Benson D. W. Jr. Gelb B. D. Giglia T. M. Goldmuntz E. (2007). American heart association congenital cardiac defects committee CoCDitY: **Gengtic basis for congenital heart defects: current knowledge: a scientific statement from the American heart association congenital cardiac defects committee, council on cardiovascular disease in the young: endorsed by the American academy of Pediatrics** . Circulation 115, 3015–3038. 10.1161/CIRCULATIONAHA.106.183056 17519398

[B117] Potdar N. Singh R. Mistry V. Evans M. D. Farmer P. B. Konje J. C. (2009). First-trimester increase in oxidative stress and risk of small-for-gestational-age fetus. BJOG 116, 637–642. 10.1111/j.1471-0528.2008.02096.x 19298438

[B118] Pulgaron E. R. Delamater A. M. (2014). Obesity and type 2 diabetes in children: epidemiology and treatment. Curr. Diab Rep. 14, 508. 10.1007/s11892-014-0508-y 24919749 PMC4099943

[B119] Quintero R. A. (2003). Twin-twin transfusion syndrome. Clin. Perinatol. 30, 591–600. 10.1016/s0095-5108(03)00051-4 14533898

[B120] Raijmakers M. T. Peters W. H. Steegers E. A. Poston L. (2004). NAD(P)H oxidase associated superoxide production in human placenta from normotensive and pre-eclamptic women. Placenta 25 (Suppl. A), S85–S89. 10.1016/j.placenta.2004.01.009 15033313

[B121] Ranke M. B. Wit J. M. (2018). Growth hormone - past, present and future. Nat. Rev. Endocrinol. 14, 285–300. 10.1038/nrendo.2018.22 29546874

[B122] Rivera J. C. Dabouz R. Noueihed B. Omri S. Tahiri H. Chemtob S. (2017). Ischemic retinopathies: oxidative stress and inflammation. Oxid. Med. Cell Longev. 2017, 3940241. 10.1155/2017/3940241 29410732 PMC5749295

[B123] Rouhier N. Lemaire S. D. Jacquot J. P. (2008). The role of glutathione in photosynthetic organisms: emerging functions for glutaredoxins and glutathionylation. Annu. Rev. Plant Biol. 59, 143–166. 10.1146/annurev.arplant.59.032607.092811 18444899

[B124] Rychert-Stos M. Walczak M. Horodnicka-Jozwa A. Romanowska H. Katuszonek D. Wyka K. (2022). Do trace elements influence the course of newly diagnosed type 1 diabetes mellitus? Neuro Endocrinol. Lett. 43, 247–256. 36584401

[B125] Saker M. Soulimane Mokhtari N. Merzouk S. A. Merzouk H. Belarbi B. Narce M. (2008). Oxidant and antioxidant status in mothers and their newborns according to birthweight. Eur. J. Obstet. Gynecol. Reprod. Biol. 141, 95–99. 10.1016/j.ejogrb.2008.07.013 18760523

[B126] Saugstad O. D. (2001). Is oxygen more toxic than currently believed? Pediatrics 108, 1203–1205. 10.1542/peds.108.5.1203 11694702

[B127] Shtaif B. Bar-Maisels M. Gabet Y. Hiram-Bab S. Yackobovitch-Gavan M. Phillip M. (2020). Cartilage -specific knockout of Sirt1 significantly reduces bone quality and catch-up growth efficiency. Bone 138, 115468. 10.1016/j.bone.2020.115468 32512163

[B128] Sies H. Jones D. P. (2020). Reactive oxygen species (ROS) as pleiotropic physiological signalling agents. Nat. Rev. Mol. Cell Biol. 21, 363–383. 10.1038/s41580-020-0230-3 32231263

[B129] Singleton P. A. Pendyala S. Gorshkova I. A. Mambetsariev N. Moitra J. Garcia J. G. (2009). Dynamin 2 and c-Abl are novel regulators of hyperoxia-mediated NADPH oxidase activation and reactive oxygen species production in caveolin-enriched microdomains of the endothelium. J. Biol. Chem. 284, 34964–34975. 10.1074/jbc.M109.013771 19833721 PMC2787359

[B130] Sinha B. Wu Q. Li W. Tu Y. Sirianni A. C. Chen Y. (2018). Protection of melatonin in experimental models of newborn hypoxic-ischemic brain injury through MT1 receptor. J. Pineal Res. 64. 10.1111/jpi.12443 28796402

[B131] Steinhorn R. H. Albert G. Swartz D. D. Russell J. A. Levine C. R. Davis J. M. (2001). Recombinant human superoxide dismutase enhances the effect of inhaled nitric oxide in persistent pulmonary hypertension. Am. J. Respir. Crit. Care Med. 164, 834–839. 10.1164/ajrccm.164.5.2010104 11549542

[B132] Stenzel A. P. Carvalho R. Jesus P. Bull A. Pereira S. Saboya C. (2018). Serum antioxidant associations with metabolic characteristics in metabolically healthy and unhealthy adolescents with severe obesity: an observational study. Nutrients 10, 150. 10.3390/nu10020150 29385682 PMC5852726

[B133] Thompson A. M. Bizzarro M. J. (2008). Necrotizing enterocolitis in newborns: pathogenesis, prevention and management. Drugs 68, 1227–1238. 10.2165/00003495-200868090-00004 18547133

[B134] Tordjman S. Chokron S. Delorme R. Charrier A. Bellissant E. Jaafari N. (2017). Melatonin: pharmacology, functions and therapeutic benefits. Curr. Neuropharmacol. 15, 434–443. 10.2174/1570159X14666161228122115 28503116 PMC5405617

[B135] Tran K. D. Cernichiaro-Espinosa L. A. Berrocal A. M. (2018). Management of retinopathy of Prematurity--Use of Anti-VEGF therapy. Asia Pac J. Ophthalmol. (Phila) 7, 56–62. 10.22608/APO.2017436 29376233

[B136] Trocino R. A. Akazawa S. Ishibashi M. Matsumoto K. Matsuo H. Yamamoto H. (1995). Significance of glutathione depletion and oxidative stress in early embryogenesis in glucose-induced rat embryo culture. Diabetes 44, 992–998. 10.2337/diab.44.8.992 7622006

[B137] Tsang J. K. W. Liu J. Lo A. C. Y. (2019). Vascular and neuronal protection in the developing retina: potential therapeutic targets for retinopathy of prematurity. Int. J. Mol. Sci. 20, 4321. 10.3390/ijms20174321 31484463 PMC6747312

[B138] Underwood M. A. (2012). Paneth cells and necrotizing enterocolitis. Gut Microbes 3, 562–565. 10.4161/gmic.21738 22895084 PMC3495794

[B139] Valko M. Leibfritz D. Moncol J. Cronin M. T. Mazur M. Telser J. (2007). Free radicals and antioxidants in normal physiological functions and human disease. Int. J. Biochem. Cell Biol. 39, 44–84. 10.1016/j.biocel.2006.07.001 16978905

[B140] van Velthoven C. T. Gonzalez F. Vexler Z. S. Ferriero D. M. (2014). Stem cells for neonatal stroke-the future is here. Front. Cell Neurosci. 8, 207. 10.3389/fncel.2014.00207 25120432 PMC4110439

[B141] Vento M. Moro M. Escrig R. Arruza L. Villar G. Izquierdo I. (2009). Preterm resuscitation with low oxygen causes less oxidative stress, inflammation, and chronic lung disease. Pediatrics 124, e439–e449. 10.1542/peds.2009-0434 19661049

[B142] Victor V. M. Rocha M. Herance R. Hernandez-Mijares A. (2011). Oxidative stress and mitochondrial dysfunction in type 2 diabetes. Curr. Pharm. Des. 17, 3947–3958. 10.2174/138161211798764915 22188447

[B143] Vinukonda G. Csiszar A. Hu F. Dummula K. Pandey N. K. Zia M. T. (2010). Neuroprotection in a rabbit model of intraventricular haemorrhage by cyclooxygenase-2, prostanoid receptor-1 or tumour necrosis factor-alpha inhibition. Brain 133, 2264–2280. 10.1093/brain/awq107 20488889 PMC3139931

[B144] Wang F. Fisher S. A. Zhong J. Wu Y. Yang P. (2015a). Superoxide dismutase 1 *in vivo* ameliorates maternal diabetes mellitus-induced apoptosis and heart defects through restoration of impaired wnt signaling. Circ. Cardiovasc Genet. 8, 665–676. 10.1161/CIRCGENETICS.115.001138 26232087 PMC4618088

[B145] Wang F. Reece E. A. Yang P. (2015b). Oxidative stress is responsible for maternal diabetes-impaired transforming growth factor beta signaling in the developing mouse heart. Am. J. Obstet. Gynecol. 212, 650 e651–11. 10.1016/j.ajog.2015.01.014 PMC4417061 25595579

[B146] Wang H. Zhang S. X. Hartnett M. E. (2013). Signaling pathways triggered by oxidative stress that mediate features of severe retinopathy of prematurity. JAMA Ophthalmol. 131, 80–85. 10.1001/jamaophthalmol.2013.986 23307212 PMC3703446

[B147] Wang H. C. Chou H. C. Chen C. M. (2023). Molecular mechanisms of hyperoxia-induced neonatal intestinal injury. Int. J. Mol. Sci. 24, 4366. 10.3390/ijms24054366 36901800 PMC10002283

[B148] Wang X. (2009). The antiapoptotic activity of melatonin in neurodegenerative diseases. CNS Neurosci. Ther. 15, 345–357. 10.1111/j.1755-5949.2009.00105.x 19818070 PMC2846661

[B149] Wang X. Wang Y. Kim H. P. Nakahira K. Ryter S. W. Choi A. M. (2007). Carbon monoxide protects against hyperoxia-induced endothelial cell apoptosis by inhibiting reactive oxygen species formation. J. Biol. Chem. 282, 1718–1726. 10.1074/jbc.M607610200 17135272

[B150] Watts J. L. Milner R. Zipursky A. Paes B. Ling E. Gill G. (1991). Failure of supplementation with vitamin E to prevent bronchopulmonary dysplasia in infants less than 1,500 g birth weight. Eur. Respir. J. 4, 188–190. 10.1183/09031936.93.04020188 2044736

[B151] Yamamoto M. Takahashi Y. (2018). The essential role of SIRT1 in hypothalamic-pituitary axis. Front. Endocrinol. (Lausanne) 9, 605. 10.3389/fendo.2018.00605 30405528 PMC6205959

[B152] Yang P. Zhao Z. Reece E. A. (2008). Activation of oxidative stress signaling that is implicated in apoptosis with a mouse model of diabetic embryopathy. Am. J. Obstet. Gynecol. 198, 130 e131–e7. 10.1016/j.ajog.2007.06.070 18166327

[B153] Zhao J. Le M. Li J. Huang Q. Chen H. Zhang W. (2023). LINC00938 alleviates hypoxia ischemia encephalopathy induced neonatal brain injury by regulating oxidative stress and inhibiting JNK/p38 MAPK signaling pathway. Exp. Neurol. 367, 114449. 10.1016/j.expneurol.2023.114449 37257715

[B154] Zhao M. Tang S. Xin J. Wei Y. Liu D. (2018). Reactive oxygen species induce injury of the intestinal epithelium during hyperoxia. Int. J. Mol. Med. 41, 322–330. 10.3892/ijmm.2017.3247 29138796 PMC5746288

[B155] Zhu J. J. Yu B. Y. Fu C. C. He M. Z. Zhu J. H. Chen B. W. (2020). LXA4 protects against hypoxic-ischemic damage in neonatal rats by reducing the inflammatory response via the IκB/NF-κB pathway. Int. Immunopharmacol. 89, 107095. 10.1016/j.intimp.2020.107095 33096360

[B156] Zhu L. Liu Y. P. Huang Y. T. Zhou Z. J. Liu J. F. Yu L. M. (2024a). Cellular and molecular biology of posttranslational modifications in cardiovascular disease. Biomed. Pharmacother. 179, 117374. 10.1016/j.biopha.2024.117374 39217836

[B157] Zhu L. Ruan X. Wang J. Yan Y. Tang C. Xu Y. (2024b). Higher oxidative balance score is linearly associated with reduced prevalence of chronic kidney disease in individuals with metabolic syndrome: evidence from NHANES 1999-2018. Front. Nutr. 11, 1442274. 10.3389/fnut.2024.1442274 39403399 PMC11472227

[B158] Zia M. T. Csiszar A. Labinskyy N. Hu F. Vinukonda G. LaGamma E. F. (2009). Oxidative-nitrosative stress in a rabbit pup model of germinal matrix hemorrhage: role of NAD(P)H oxidase. Stroke 40, 2191–2198. 10.1161/STROKEAHA.108.544759 19372442 PMC2726799

[B159] Zuo H. Jiang W. Gao J. Ma Z. Li C. Peng Y. (2025). SYISL knockout promotes embryonic muscle development of offspring by modulating maternal gut microbiota and fetal myogenic cell dynamics. Adv. Sci. (Weinh) 12, e2410953. 10.1002/advs.202410953 39680624 PMC11809340

[B160] Zysk M. Gapys B. Ronowska A. Gul-Hinc S. Erlandsson A. Iwanicki A. (2018). Protective effects of voltage-gated calcium channel antagonists against zinc toxicity in SN56 neuroblastoma cholinergic cells. PLoS One 13, e0209363. 10.1371/journal.pone.0209363 30571745 PMC6301650

